# Historical range, extirpation and prospects for reintroduction of saigas in China

**DOI:** 10.1038/srep44200

**Published:** 2017-03-09

**Authors:** Shaopeng Cui, E. J. Milner-Gulland, Navinder J. Singh, Hongjun Chu, Chunwang Li, Jing Chen, Zhigang Jiang

**Affiliations:** 1 Key Laboratory of Animal Ecology and Conservation Biology, Institute of Zoology, Chinese Academy of Sciences, Beijing, China; 2 University of Chinese Academy of Sciences, Beijing, China; 3 Department of Zoology, University of Oxford, South Parks Road, Oxford, United Kingdom; 4 Department of Wildlife, Fish and Environmental Studies, Swedish University of Agricultural Sciences, Umeå, Sweden; 5 College of Resources and Environment Sciences, Xinjiang University, Urumqi, Xinjiang, China; 6 Altay Management Station, Mt. Kalamaili Ungulate Nature Reserve, Altay, Xinjiang, China

## Abstract

An assessment of historical distribution patterns and potential reintroduction sites is important for reducing the risk of reintroduction failure of endangered species. The saiga antelope, *Saiga tatarica*, was extirpated in the mid-20th century in China. A captive population was established in the Wuwei Endangered Wildlife Breeding Centre (WEWBC) in the 1980s. Reintroduction is planned, but so far, no action has been taken. In this study, we delineated the historical distribution and potential reintroduction areas of saigas in China, using a literature review, interviews and predictive modelling. Results suggest that most of the seasonally suitable areas are non-overlapping, and China may have been a peripheral part of the main saiga range. WEWBC is not an ideal reintroduction site due to its low habitat suitability. Furthermore, we infer that two different movement patterns existed historically (regular migration and nomadic wandering). Our results demonstrate the challenges of restoring a free-ranging, self-sustaining saiga population in China. We recommend the setting up of additional breeding centres in protected areas within the potential saiga range in Xinjiang, and the development of a national action plan to provide a framework for the future recovery of the species.

Ungulate migrations are among the most spectacular natural phenomena on the planet^1^,^2^. These seasonal, round-trip movements of thousands of individuals at large spatial scales have important ecological consequences for the structure and functioning of many terrestrial ecosystems^2^,^3^. However, due to human activities, many migratory ungulates have become threatened or extinct during the last century^4^,^5^. Complex migratory behaviours provide a special challenge for conservation and restoration of species^1^,^2^,^6^. Such is the case for the saiga antelope (*Saiga tatarica*), a migratory species endemic to the semi-arid rangelands of Central Asia and the pre-Caspian^7^,^8^. Two saiga subspecies are recognized: *S. t. tatarica* in the Russian Federation, Kazakhstan and Uzbekistan, and *S. t. mongolica* in Mongolia^8^,^9^,^10^. The saiga used to be abundant and has been traditionally hunted for meat, horns and hides since prehistoric times^7^. However, the collapse of the Soviet Union and subsequent heavy poaching led to a 95% reduction in the species’ population size in less than 10 years^8^, leading to its categorisation as “Critically Endangered” on the IUCN Red List in 2002. In recent years, with substantial conservation investment from range states and international conservation bodies, the outlook for the species has improved. In spring 2015, however, one population of saiga in Kazakhstan suffered a mass mortality event in which over 200,000 individuals died in weeks probably due to *Pasteurella multocida*^11^.

The saiga was once widely distributed in northwest China^12^,^13^. Saiga horns are claimed to have high value in traditional Chinese medicine for their perceived effectiveness for many diseases^14^, which now is one of the factors driving heavy poaching^7^,^8^. Records of the use of saiga horns in traditional Chinese medicine can be traced back to the “*Shennong’s Materia Medica*” about 2,000 years ago^14^. In the middle of the 20th century, saiga populations declined rapidly to extinction in China as a result of natural and human factors^15^,^16^. However, compared with other regions in Central Asia, the historical distribution and status of saigas in China is unclear^7^,^17^.

China started to reintroduce saiga in the 1980s, when the former Ministry of Forestry (now State Forestry Administration) established the Wuwei Endangered Wildlife Breeding Centre (WEWBC, now called Gansu Endangered Animal Protection Centre) in 1987. Eleven adult saigas from San Diego Zoo, USA, and Berlin Taie Zoo, Germany, were introduced to WEWBC to form a founder herd in 1988–1991, and one calf was added from the wild saiga population in Kalmykia (Russia) in 1997^16^. Currently, the population at WEWBC increased to over 170 individuals. However, founder events, bottlenecks and inbreeding have resulted in low genetic diversity in this captive population, which, together with harsh winter conditions and severe disease, has led to large fluctuations in population size^18^. For instance, the number of saigas at WEWBC decreased by 77% to nine individuals in 2000, which was the most dramatic crash since the founding population was established (Supplementary Fig. S1). There is also a question about the appropriateness of the genetic composition of the founder population for wild release, since the Kalmykian population is the furthest one from China, the genetic heritage of the zoo-origin animals is unclear, and there are genetic differences between saiga populations across the range^9^,^10^. Moreover, it remains controversial which subspecies of saiga was historically distributed in China. Some studies have stated that the Mongolian subspecies, *S. t. mongolica*, was found in China, either as the only subspecies, or alongside the nominate subspecies (e.g. refs 13 and 19). However, others suggest that only *S. t. tatarica* existed (e.g. refs 20 and 21).

In order to restore extirpated populations through reintroduction, a system of interrelated ecological, political and socio-economic measures for habitat monitoring and control of human disturbances is required^22^. Identifying the former range and causes of extirpation of the species in question is the first step, as this provides a basis for conservation efforts^23^. The historical range provides a geographical framework for assessing possible reintroduction sites. Likewise, determining the large-scale movement patterns of historical populations is also needed in order to reintroduce a mobile species^1^,^24^. However, due to the scarce and fragmented historical occurrence data for the species, few studies have explicitly examined its former range in China or developed a timeline of its decline^12^,^13^,^17^. Furthermore, we are not aware of any study that has determined how historical saiga populations moved seasonally between China and neighbouring countries and whether the current habitat conditions can sustain reintroduced populations. This is vital information for any reintroduction programme.

Thus, our aim was firstly to collate a range of datasets in order to delineate the historical distribution and chart the extirpation of the saiga in China. Secondly, we built species distribution models (SDMs) based on information on saiga habitat use in neighbouring parts of Kazakhstan to predict the potential suitable areas for future saiga reintroductions. Finally, we discuss the challenges concerning the persistence of captive and wild saiga populations in China.

## Results

### Historical distribution of saiga in China

**Figure 1:**
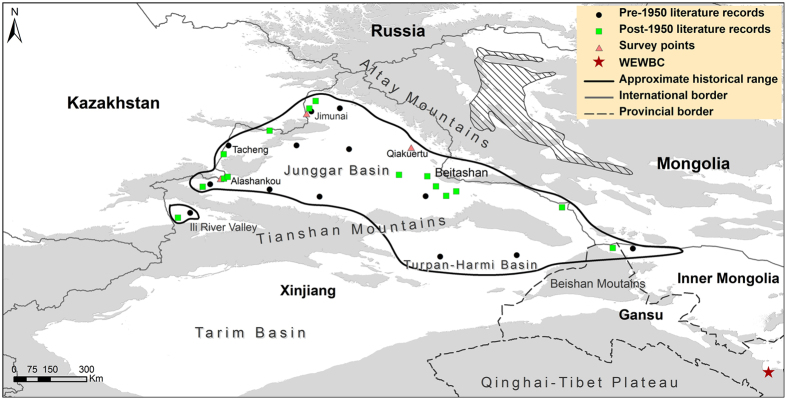
Historical distribution of the saiga in China. The historical range of Mongolian saiga (hatched area) is adapted from Lhagvasuren *et al*.^30^. Grey represents mountains at altitude over 1500 m, which form effective barriers to saiga movement. Notice that the WEWBC (Wuwei Endangered Wildlife Breeding Centre) is well outside the saiga’s range. This figure is generated based on digital elevation data from the CGIAR International Research Centers (http://srtm.csi.cgiar.org/) using ArcGIS 9.3 (ESRI, Redland, CA, USA, http://www.esri.com/).

We found a total of 28 historical records, which covered the time period from the end of the 19th century to the 1950s (Supplementary Table S1). Many of the references described the saiga distribution roughly (e.g. Przewalski^25^ stated that saiga occurred in the west of the Junggar (Dzungarian) Basin). We used all the records to map the approximate historical range of the species (Fig. 1). Prior to 1950, 13 records were identified in northwest China and saigas covered a wide geographical range, from the border areas between China and Kazakhstan, through the Junggar and Turpan-Hami Basins, to the northern Beishan Mountains and western Inner Mongolia. Also, the saiga was recorded in the Ili river valley located the southwest of Tianshan Mountains. Fifteen records were found for the first 10 years after the founding of the People’s Republic of China (PRC) in 1949. The saiga distribution was gradually reduced to the China-Kazakhstan and China-Mongolia border areas, including Alashankou, Tacheng, Jimunai, Ili river valley, and northern Beitashan Mountains. We found no literature records from the 1960s or later. The documented historical range was completely separated from that of the Mongolian saiga by the Altay Mountains. Moreover, WEWBC was outside of the historical range (Fig. 1).

The interview results supported the literature review. In Qiakuertu, all of the ten local herders interviewed said that they had not seen saigas since the 1950s, whereas six said the species had been seen in Junggar Basin by their elders (especially fathers) and two still remembered the high price of saiga horns at that time. In Alashankou and Jimunai, both local herders and reserve staff said that saigas were seen during the 1950s. However, most of the local herders were not sure about which season saigas were seen in, because of the long time which had elapsed since their observations. In Qiakuertu, some local herders said that saigas were once found in the winter pastures (i.e. Junggar Basin) by their fathers. All interviewees thought that the overhunting for meat and horns accounted for the extirpation of the species. A staff member of Xia’erxili Nature Reserve said that a few vagrant saigas from Betpak-dala region were once reported by local herders around Alashankou. He stated that the increased availability of semi-automatic firearms and vehicles facilitated the large-scale hunting of saigas around Alashankou in the summers of the late 1950s.

### Model performance, explanatory variables and the predicted distribution

**Figure 2:**
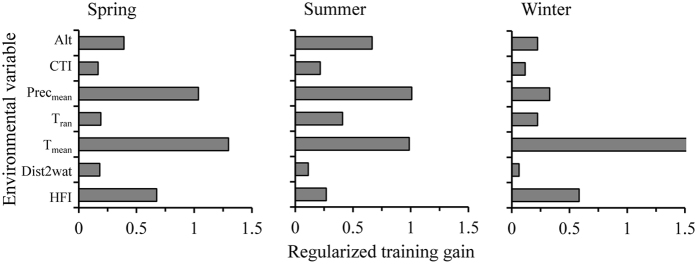
Jackknife analyses of the importance of environmental variables when developing species distribution models in different seasons. For each variable, the bars show the regularized gain achieved with training data of variable used in isolation. CTI, Compound Topographic Index; Prec_mean_, average monthly mean precipitation; T_ran_, mean monthly temperature range; T_mean_, average monthly mean temperature; Dist2wat, distance to the nearest water; HFI, Human Footprint Index.

Models were built to predict saiga habitat use within the current range of the neighbouring extant population (Betpak-dala, Kazakhstan) and the known historical range in China, and tested against data on saiga presence/absence. The AUC (area under the receiver operating characteristic curve) values revealed that the models performed well and provided reasonable discrimination. The training and testing AUC were 0.967 ± 0.001 SD and 0.958 ± 0.014 for spring, 0.966 ± 0.002 and 0.955 ± 0.015 for summer, and 0.965 ± 0.001 and 0.954 ± 0.017 for winter, respectively. The jackknife test of variable importance (Fig. 2) showed that the average monthly mean temperature (T_mean_), average monthly mean precipitation (Prec_mean_), mean monthly temperature range (T_ran_), and altitude (Alt) gave the most useful information (i.e. the highest gain when used in isolation). By contrast, the Compound Topographic Index (CTI, also known as the topographic wetness index) and distance to the nearest water source (Dist2wat) made relatively small contributions to the model. The Human Footprint Index (HFI) was an effective predictor of potentially suitable areas of saiga range in China, especially in spring and winter, suggesting that post-1950s land use transformation may have had an important impact on current habitat suitability for saigas (Fig. 2).

**Figure 3:**
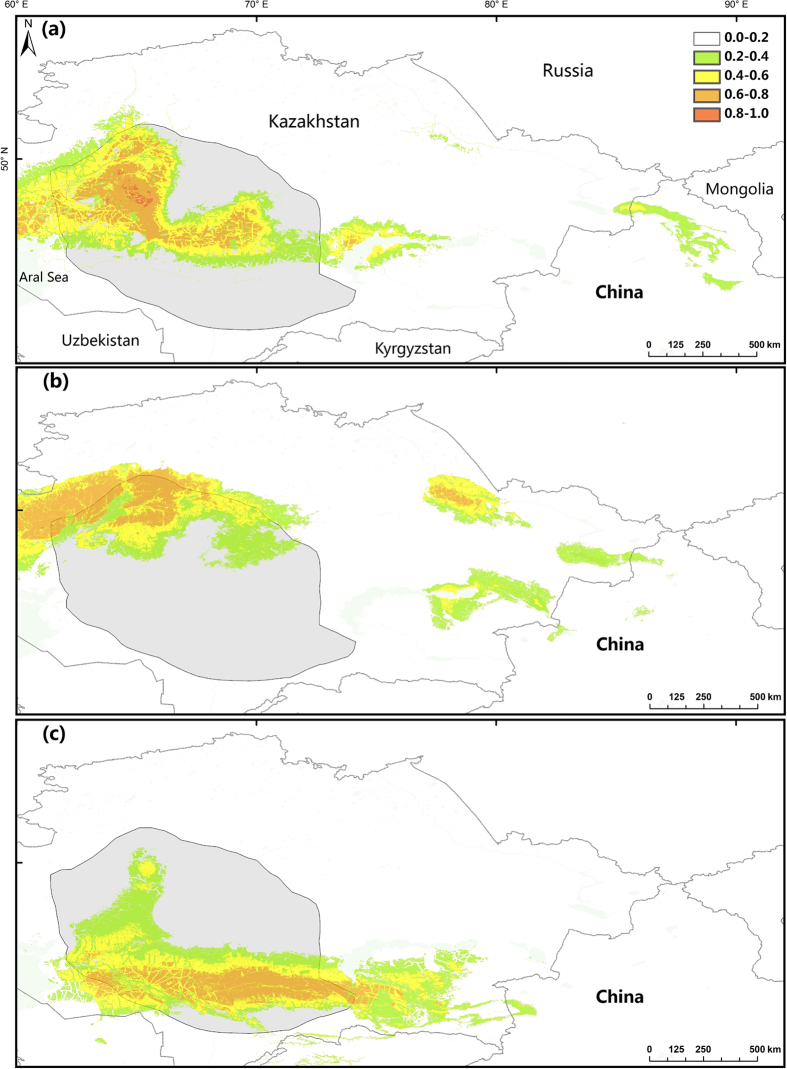
Predicted probability distribution maps of saiga in spring (**a**), summer (**b**), and winter (**c**). The current range of the Betpak-dala saiga population (grey) is displayed following Milner-Gulland *et al*.^8^. Values for the probability of habitat use range from 0 (lowest probability) to 1 (highest probability). The map is generated based on the projected distributions of saiga for each season using ArcGIS 9.3 (ESRI, Redland, CA, USA, http://www.esri.com/).

Most areas of the study region were predicted to have low suitability as saiga habitat, with over 95% of the area modelled having a probability of occupancy of <0.5 (Table 1). More suitable areas occurred mainly within the current Betpak-dala population ranges (Fig. 3; Table 1).

The highest suitability areas predicted from the models matched well with the known presence records. However, the areas around Balkhash Lake were predicted to contain potential habitat in all three seasons, in contrast to the saiga’s actual presence, which is mostly in winter. In spring, highly suitable ranges occurred in the central and western parts of the area, while in summer, the potential habitat was to the east. Additionally, away from the current Betpak-dala population ranges, there was a potentially suitable area around the Irtysh River in the northeast of Kazakhstan, bordering Russia. In winter, the potential range was larger than the currently occupied range, extending to the southern part of Lake Balkhash (Fig. 3).

### Potential reintroduction sites for saigas in China

**Figure 4:**
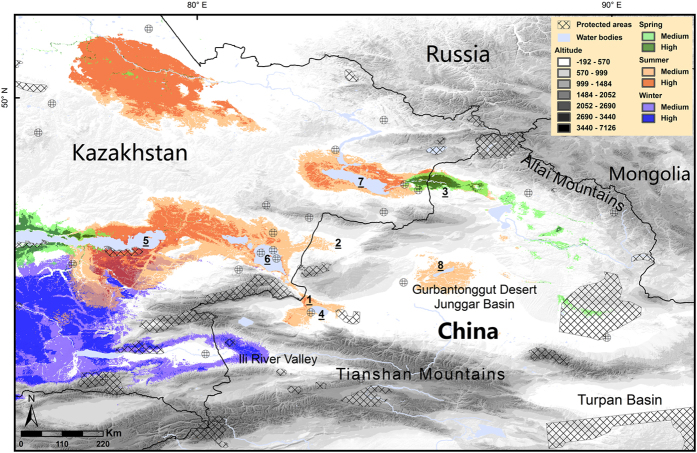
Predicted habitat suitability for saigas in different seasons in China. The suitability is defined as the probability of occupancy and we used two thresholds to divide the predictions into medium- and high-suitability areas (see Methods for details). The numbers indicate locations mentioned in the text: 1, Alashankou; 2, Tacheng; 3, Jimunai-Habahe; 4, Aibi Lake; 5, Balkhash Lake; 6, Alakol Lake; 7, Zaysan Lake; 8, Manas Lake. The figure is generated based on the projected distributions of saiga for each season, digital elevation data from the CGIAR International Research Centers (http://srtm.csi.cgiar.org/), and nature reserve boundaries from the World Database on Protected Areas (http://www.protectedplanet.net/) using ArcGIS 9.3 (ESRI, Redland, CA, USA, http://www.esri.com/).

The predictive modelling identified five regions containing potentially suitable habitat for saigas in China; areas surrounding Aibi Lake, the Jimunai-Habahe border areas, Tacheng, Junggar Basin and the Ili river valley (Fig. 4). The areas of predicted suitable habitats were very small, especially the potential wintering range, which had the smallest area (5,100 km^2^). The areas with high suitability in each season were all below 2,000 km^2^ (Table 1). The current potentially suitable range was substantially smaller than the historical distribution in China. Most of the predicted suitable ranges were non-overlapping in different seasons, which is problematic for reintroductions, though a distinct overlap of the potential spring and summer areas in Jimunai-Habahe region was observed. The overlapping region was connected by the Irtysh River with the Zaysan Lake of Kazakhstan, around which medium-to-high suitability habitats were also identified in spring and summer. In summer, potentially suitable areas occurred around Balkhash Lake in Kazakhstan, extending east to Aibi Lake over Alakol Lake and Alashankou. Additionally, the areas around Manas Lake in Junggar Basin were predicted to have medium suitability. Some scattered potential spring areas were mainly located to the north and east of Junggar Basin. Suitable wintering habitat was predicted only in the Ili river valley near the border with Kazakhstan, which was isolated from potential spring and summer areas by the Tianshan Mountains (Fig. 4).

The Alashankou, Tacheng, Jimunai-Habahe and Ili river valley could potentially form migratory corridors, in accordance with the results of Liang^13^ (Fig. 4). Five nature reserves were identified as potential reintroduction sites in China, based upon their locations within one or more areas predicted to be seasonally suitable. These were Mt. Kalamaili Ungulate Nature Reserve, Xia’erxili Nature Reserve, Ganjiahu Haloxylon Forest National Nature Reserve, Aibi Lake Wetland National Nature Reserve, and Huocheng Sizhaolugui Nature Reserve.

### A comparison of environmental variables between range areas

**Figure 5:**
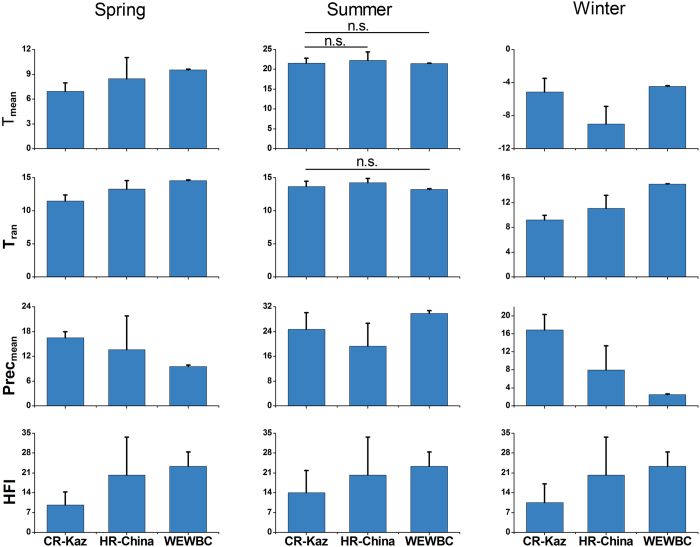
Comparison of environmental variables in different seasons between the current Betpak-dala population range in Kazakhstan (CR-Kaz), historical range in China (HR-China), and the captive breeding centre (WEWBC). All data are presented as mean + SD, and all variables are significantly different by Kruskal-Wallis tests at *P* < 0.05, except where n.s. indicates no significant difference by post-hoc Dunn’s multiple comparisons test (*P* > 0.05). Abbreviations follow Fig. 2. Temperature is in °C, precipitation in mm.

A comparison of the three bioclimatic variables and the HFI between our three areas of interest (the known distribution range in Betpak-dala, Kazakhstan, the historical range in China, and WEWBC) showed significant differences between areas in most seasons. The only exceptions were that there were no significant differences in T_mean_ and T_ran_ between Kazakhstan and WEWBC in summer (Dunn’s post-hoc multiple comparison tests; T_mean_: *P* = 0.432; T_ran_: *P* = 0.054), and T_mean_ between Kazakhstan and China in summer (*P* = 0.052, Fig. 5).

## Discussion

### Historical distribution and movement patterns of saigas

Historically, saiga populations were generally thought to be distributed in the Junggar Basin, but other historical ranges (especially the Ili river valley) were less well recorded^19^,^26^. Our results revealed a substantial difference in the recorded locations in the pre-1950 period compared to post-1950. This may represent an observer bias, or genuine changes in saiga range. However, as shown by previous authors^13^, after 1950, saigas were mainly confined to the border areas, especially with Kazakhstan. It is reasonable to assume that the species had already suffered substantial anthropogenic disturbance in the mid-20th century^4^,^15^. During and after the 1960s, although several large-scale field surveys were conducted in Xinjiang, only a few saiga skulls were found^27^.

The factors leading to the extirpation of saigas in China include overhunting, habitat reduction, and fragmentation of migratory routes by the closure of borders^27^. Since the 1950s, the settlement of retired soldiers and civilians led to large-scale land reclamation and reduced wildlife habitat in Xinjiang^15^,^26^. In the 1930s, 50,000 pairs of saiga horns were sold in the traditional medicine market within a single year^27^. Similarly high levels of hunting and trade took place in Kazakhstan during the late 19th century and the early 20th century, leaving that population at very low levels from which it only recovered once strict rules were introduced after the Soviet revolution^7^. In the 1950s, border fences between China and neighbouring countries were erected in Xinjiang, blocking the saiga’s movement routes^26^, at a time when populations were recovering in Kazakhstan and could potentially have recolonized China^7^. Similar processes affected other species in Central Asia, such as Przewalski’s horse *Equus ferus przewalskii*, wild camel *Camelus ferus* and Sino-Mongolian beaver *Castor fiber*. These species were disproportionately threatened during the latter half of the 20th century^15^, due principally to overhunting and habitat loss/degradation, coinciding with widespread social and economic changes across the region^4^.

The populations from which individuals are used for reintroduction should be as genetically close to the native population for more robust adaptation to the ecological characteristics of reintroduction areas^22^,^28^. We argue that only *S. t. tatarica* occurred in China. Historically, both subspecies were recorded in Mongolia, however since 1960s, the nominate subspecies has not been observed^29^,^30^. The nominate subspecies once lived in the Dzungarian Gobi which borders China, whereas the Mongolian subspecies only occurred in Mongolia, ranging from the Uvs Nuur Basin, southeastwards through the Great Lakes Basin to the Khuisiin Gobi and Shargyn Gobi (Fig. 1)^30^,^31^. The historical saiga range in China is adjacent to the Dzungarian Gobi but completely separated from that of Mongolian saiga by the Altay Mountains^29^. Conversely, the historical population can mix with the Kazakh populations by migrating to the border areas^13^. Therefore, the appropriate source population for re-establishing the species in China would be the Betpak-dala population rather than the Mongolian subspecies. Indeed, some saiga concentrations have been recently reported in the northeastern Pribalkhash and southern Balkhash region of Kazakhstan, southeast of the currently accepted range of the Kazakhstan population, and close to China^32^.

In previous studies, Liang^13^ and Wang^26^ stated that the species was distributed extensively but not continuously in China. Taken together with our results, this suggests that China may have had several saiga populations. Our interview surveys showed that saiga occurred historically in the Junggar Basin in winter, whereas the models predicted no suitable wintering range in the basin. The western Junggar Boundary Mountains and Tianshan Mountains (Fig. 1) create a barrier effect that not only restricts the movement of the species, but also blocks the eastward penetration of the Atlantic westerlies. Together with the influence of Mongolia’s arid climate, it leads to an increasingly continental climate and decreasing precipitation from Junggar Basin to the Mongolian Gobi^33^,^34^. The different climate and associated resource availability could not meet the need for a regular migration. Based on these results we infer that there were historically two movement patterns; a regular migration in the border areas of China and Kazakhstan (e.g. Jimunai-Habahe, Tacheng and Ili river valley), potentially integrated with wider movement patterns of the Betpak-dala population, and nomadic movements within the Junggar Basin and its eastern areas, similar to those found in the Mongolian population. These nomadic movements could have led to some connectivity between populations in the China-Mongolia border areas.

### Locations for saiga reintroduction

Our results indicate that China mainly provides peripheral, rather than representative, habitats for current saiga populations. Only small, isolated areas remain suitable for future reintroductions. This implies that, as previous reports have shown^13^, in order to establish a sustainable population naturally, saigas would need to migrate from Kazakhstan into China. This need for trans-boundary migration makes it particularly difficult to manage the species and its habitat effectively^35^,^36^. Border fences are a serious movement barrier for saigas, as has been recently demonstrated by the erection of a border fence on the Ustyurt Plateau between Kazakhstan and Uzbekistan, disrupting the migration of the Ustyurt population^35^,^37^. Establishing transboundary nature reserves requires suitable political and socio-economic conditions, and is usually not easy to achieve^36^. Therefore if saigas were to establish in China, it is not possible to rely on natural recolonization.

Most of the bioclimatic variables were significantly different between the former saiga range in China and the current Betpak-dala population ranges, especially in winter (Fig. 5). Given this, the small initial size of a reintroduced population, and the challenges of most areas not being suitable all year round, it may be difficult for saigas to establish in China. Singh and Milner-Gulland^6^ emphasized that climate change interacting with human disturbance can greatly influence saiga distributions during the calving season^38^,^39^. A small saiga population in limited habitat would be unable to escape harsh climatic conditions (e.g. so-called *dzhut*) and winter resources may be limited. Therefore, supplemental feeding may be necessary^40^.

The ultimate aim of any reintroduction program is to establish a viable, free-ranging population in the wild^23^. The study of movement patterns can provide insights into how mobile species respond to threats and environmental conditions^24^,^36^. Better understanding of the mechanisms underlying movement patterns at multiple scales is therefore necessary to assess the likely success of reintroduction^39^. Some saiga populations have already had their movement curtailed (e.g. the pre-Caspian population), or inhabit the same geographic regions throughout the year (e.g. Vozrozhdenie peninsula in Uzbekistan)^39^. These confined or isolated groups appear able to survive in small numbers, which may provide a valuable opportunity to examine the influence of variations in movement patterns on population viability.

### The way forward for saiga reintroduction in China

Reintroduction success is strongly related to the number of released animals^41^. Considering the combined effects of deterministic and stochastic factors, a self-sustaining captive population (enough breeding stock and high genetic diversity) is regarded as the foundation of a successful reintroduction^42^. Nowadays only WEWBC keeps a captive population in China, but its population size and genetic diversity levels are inadequate for reintroduction^18^. Therefore, the translocation of more saigas from wild populations, as well as improved management at WEWBC, is imperative if reintroduction is to succeed. Also, it is important to follow IUCN’s reintroduction guidelines which provide a detailed and practical framework for any stage of reintroductions^23^.

Most of the failures in previous introductions of saigas were due to physiological stress^16^. More research is needed on capture strategies, immobilization procedures and transportation. Saigas are known to be particularly sensitive to stress and difficult to breed in captivity, and neither capture in order to hold saigas in captive populations nor release into the wild have been particularly successful in the past^43^. However, recent work on capture techniques to fit satellite collars has produced a protocol which appears successful in minimising stress^44^, and experience is growing on the successful breeding of saigas in captivity. Collaborative work with other captive breeding centres should be further facilitated and experiences of saiga breeding shared, enabling joint efforts through inter-regional cooperation.

Although WEWBC has bred saiga for nearly 30 years, Liu^45^ as well as Liu and Chen^46^ evaluated the vegetation characteristics of the centre and found that WEWBC lacked halophytes and had limited food resources in the non-growing season (October to March). Saigas in WEWBC depend heavily on human assistance (e.g. increasing water supply during the breeding season, supplementing hay in winter^45^). Additionally, as shown by former introductions, the long distance between Kazakhstan and Wuwei greatly increases the difficulty of transportation. Therefore we recommend the setting up of additional breeding centres for supporting reintroduction efforts in the potential and historical ranges of saiga, such as in Mt. Kalamaili Nature Reserve and Xia’erxili Nature Reserve. The former has had successful reintroduction experiences with Przewalski’s horse, while the latter is representative of highest habitat suitability for saiga.

### Recommendations

Throughout its history, saigas have suffered large-scale die-offs and since 1955, there have been many die-offs recorded in Kazakhstan, usually when females form large aggregations to calve in spring^47^. In May 2015, nearly 70% of the total population of saiga, over 200,000 individuals, died within two weeks. This, alongwith continued illegal hunting throughout its range, suggests that establishing one or more saiga populations in China would be a useful precautionary measure^11^.

The biological characteristics and population history of the saiga suggest that the species is remarkably resilient and can quickly recover, but this requires concerted conservation and management efforts from all the range states and the international community. The long history of comprehensive studies of saiga ecology in Russia and Kazakhstan forms the foundation of many conservation projects. China should actively participate in these projects and enhance international cooperation towards the recovery of the species. It is essential to share experiences of developing practical management measures, including wildlife-friendly fences, migration path design, and public awareness activities, so as not to repeat the mistakes and mismanagement that have occurred in the past. An action plan in China should be formulated with the support of conservation NGOs and international agreements (e.g. the Convention on Migratory Species), which can be used as a framework for designing the activities of the relevant departments.

## Methods

### Literature review

We carried out an extensive literature search in order to reconstruct the historical distribution of the saiga. Records of the species’ occurrence were obtained from a variety of primary and secondary sources, including expedition records, local chorographies, museum specimen label data, survey reports, faunal atlases and scientific literature.

Early studies of mammals in arid areas of Central Asia can be traced to the expeditions by explorers such as Przewalski from the end of the 19th century to the first half of the 20th century (Supplementary Table S1). These records are only brief, isolated accounts during expeditions, mostly of ungulate sightings in the wild. We searched mainly in the Digital Archive of Toyo Bunko Rare Books, an important sub-project of Digital Silk Road^48^. This digital archive contains 203 rare books by 76 authors. References to saigas were also found in local chorographies compiled during the late Qing Dynasty (the second half of the 19th Century). Since the founding of PRC in 1949, intensive field expeditions were carried out in western China, including the Xinjiang Integrated Scientific Expedition from 1958 to 1963, Qinghai-Tibetan Plateau Expeditions from 1975 to 1979, and surveys organized by local governments. Thus we pooled literature records roughly into two periods: pre-1950 and post-1950. Museum records were examined on the National Specimen Information Infrastructure of China^49^. Additional information was gleaned from the China Species Information System^50^, which includes a searchable database that returns a species’ natural range (including distribution maps). We also searched web databases, such as Google Scholar, Baidu Scholar, China National Knowledge Infrastructure, for available information on *Saiga tatarica*.

The historical occurrence data were quality-checked based on how detailed and biologically correct the source’s description of the presence of saiga was and our own surveys in the region. Dubious outlying records were removed. The resulting dataset was of not sufficient quality for producing predictive SDMs owing to low geographic precision. Therefore, those data were used to delineate the general distribution pattern of saigas in China.

### Interview survey

Local people often hold substantial ecological knowledge, which can be used to inform species management and policy-making. As a complementary approach, we carried out an interview survey in the Junggar Basin and China-Kazakhstan border areas of Xinjiang from July to August 2013. The Junggar Basin, characterized by a vast temperate desert-steppe ecosystem, is home to several threatened or endangered nomadic ungulates such as reintroduced Przewalski’s horse, Asiatic wild ass (*Equus hemionus*), and goitered gazelle (*Gazella subgutturosa*), but also to a traditional livestock herding culture, with transhumance between summer pasture in the Altay Mountains and winter pasture in the basin. Based on the historical range from our literature search, we chose Qiakuertu, Jimunai and Alashankou as survey sites (Fig. 1). We asked people whether, when, and at what season they had ever seen or heard saiga on their lands in the past and what factors they thought had led to the extinction of the species. We chose to interview local herders over 60, because they are most likely to possess the information concerning the fate of the species. A total of 30 local herders participated in these interviews. Furthermore, we interviewed a staff member from Xia’erxili Nature Reserve near Alashankou, which located on the China-Kazakhstan border and was historically a main migration corridor for saigas^13^.

### Species distribution model and saiga distribution data

SDMs are empirical models that combine field observations with environmental predictor variables, based on statistically or theoretically derived response surfaces^51^,^52^. They can perform well in characterizing the natural distributions of species and has become an increasingly important tool for the conservation and management of endangered species^53^,^54^,^55^. We predicted the potential saiga range using the Maximum Entropy Method (Maxent, version 3.3.3 k). Maxent is a general-purpose machine learning method for presence-only data^52^,^56^. It finds the probability distribution of maximum entropy (i.e., that is most spread out, or closest to uniform), subject to a set of constraints that represent the incomplete information about the target distribution^52^. Maxent has performed well in recent comparative studies with generally good predictive accuracy^57^.

The Betpak-dala saiga population in Kazakhstan was selected to estimate the species’ niche in the SDMs because its range provides the best indication of saiga habitat, given the lack of data availability for China. The population may have been contiguous with at least part of the range of the extinct population(s) in China^13^,^58^. We obtained saiga occurrence data in each season previously collected by Singh and Milner-Gulland^6^. With reference to Bekenov *et al*.^7^, seasons were defined as spring (March–May), summer (June–August), autumn (September–October) and winter (November–February) based on precipitation and temperature changes. Due to the lack of comparable long-term distribution data for autumn, we only made predictions for spring, summer and winter. The presence data were double-checked using spreadsheets and a geographic information system to detect duplicates and possible georeferencing errors. This yielded a final compilation of 298 records: 104 for summer, 86 for spring, and 108 for winter.

### Environmental variables

We selected seven environmental variables (describing bioclimatic features, topography, availability of water, and anthropogenic impacts) for modelling the distribution of saiga in different seasons. All variables were important determinants of saiga presence/absence identified in previous studies (Supplementary Table S2). Bioclimatic data were produced from WorldClim 1.4^59^, including the average monthly mean temperature (T_mean_), average monthly mean precipitation (Prec_mean_), and mean monthly temperature range (T_ran_). We calculated the variables for each season. Altitude (Alt) and the Compound Topographic Index (CTI) were used to represent topographic variables from the United States Geological Survey’s Hydro1K dataset^60^. We obtained water body data from the Global Lakes and Wetlands Database^61^, and calculated distance to the nearest water source (Dist2wat) for each grid cell by creating a Euclidean distance–based raster in spatial statistics tools of ArcGIS 9.3 (ESRI, Redland, USA). We used the Human Footprint Index (HFI) to incorporate anthropogenic impacts on saigas. The index is an estimate of human influence based on human settlements, land transformation, accessibility and infrastructure data^62^.

Singh *et al*.^39^ showed that vegetation productivity and precipitation variables were confounded, so we used Prec_mean_ instead of the Normalized Difference Vegetation Index (NDVI) as Prec_mean_ can act as an indicator of snow depth in winter^7^. Because environmental variables are often highly correlated, we carried out Pearson’s correlation tests across all pair-wise combinations of the selected seven environmental variables. We considered variable pairs highly correlated if |r| ≥ 0.75. The results indicated no obvious correlation. We resampled all variables to the same resolution of 30 arc-seconds using a bilinear interpolation function, which is considered to be more realistic than the nearest-neighbour method^52^.

### Model building

We implemented Maxent 3.3.3 k with mainly default settings, which have been shown to achieve good performance^56^. Additionally, we ran models with 10 cross-validated replicates and randomly assigned the presence records as training and test datasets (80 and 20%, respectively). Selection of different feature types (functions of environmental variables) was carried out automatically, following the default rules dependent on the number of presence records. We selected the logistic output format to make the model easier to interpret with suitability values ranging from 0 (the lowest suitability) to 1 (the highest suitability). Furthermore, we carried out jackknife analyses of environmental variable importance based on the regularized gain with training data of variables when used in isolation.

Different methods of defining the study region strongly affect the results of predictive modeling^63^. The range of the species over relevant periods of its history is the most appropriate extent for implementing SDMs^64^. Therefore, we defined the extent of the study region as covering the entire known range of the species in Betpak-dala and China^54^. The Betpak-dala population migrates a long distance seasonally and responds differently to environmental fluctuations in each season^39^, therefore we ran separate models for each season.

We assessed model performance using the average AUC score based on multiple replicates. AUC has been widely applied as a threshold-independent measure of model accuracy^57^,^63^. The ensemble-forecasting approach combines a number of alternative models to reach a consensus scenario and is considered to provide robust projections^65^. We obtained one final predicted distribution from the output of the ten cross-validated replicates for each season by calculating the mean suitability within each grid-cell. As different methods of threshold estimation have a great effect on the prediction map and there is still no consensus on the selection of the optimal threshold, two different thresholds were considered. MaxSS (maximum training sensitivity plus specificity) is considered to be a robust method especially when only presence data are available^66^, while TPTP (10th percentile training presence) can provide a highly conservative estimate^55^. We used the two thresholds to divide the predictions into medium- and high-suitability areas, i.e. we assumed that a grid cell is of medium suitability if its score (occurrence probability) is greater than MaxSS logistic threshold, and highly suitable if its score exceeds TPTP logistic threshold.

### Spatial and statistical analyses

Based on the areas of medium and high suitability ranges by season, we identified and extracted the potential range in China, to represent possible reintroduction sites. We then overlaid them with the existing nature reserve network to identify protected areas which could support wild saiga populations in the future. The reserve data was compiled from the World Database on Protected Areas^67^. To examine the difference in environmental variables between different ranges, we extracted and compared the values of four variables (T_mean_, T_ran_, Prec_mean_, and HFI) within different regions, including the current Betpak-dala population range in Kazakhstan (saiga occurrence records), the historical range in China (historical records), and WEWBC. Statistical analysis was performed using the Kruskal-Wallis test, followed by a post-hoc Dunn’s Multiple Comparisons test. *P* < 0.05 was regarded as statistically significant. All spatial and statistical analyses were conducted in ArcGIS 9.3 (ESRI, Redland, USA) and SPSS 19.0 (IBM, Armonk, NY, USA).

### Ethics statement

This study was reviewed and approved by the Ethical Committee of the Institute of Zoology, Chinese Academy of Sciences. All procedures performed in this study were in accordance with the instructions and permission of the Ethical Committee and the Chinese Wildlife Management Authority. All participants were informed of the study and gave informed consent.

## Additional Information

**How to cite this article:** Cui, S. *et al*. Historical range, extirpation and prospects for reintroduction of saigas in China. *Sci. Rep.* **7**, 44200; doi: 10.1038/srep44200 (2017).

**Publisher's note:** Springer Nature remains neutral with regard to jurisdictional claims in published maps and institutional affiliations.

## Supplementary Material

Supplementary Information

## Figures and Tables

**Table 1:** Predicted potential saiga habitat (unit: 10^3^ km^2^) for saiga in the whole modelled region and in China (a subset of the whole region) in different seasons.

Region and season	Medium-suitability	High-suitability	Total
Whole region			
Spring	90 (2.36)	270 (7.06)	360 (9.42)
Summer	150 (3.94)	280 (7.28)	428 (11.22)
Winter	150 (4.05)	330 (8.75)	489 (12.80)
China			
Spring	6.4 (0.17)	1.9 (0.05)	8.3 (0.22)
Summer	13.9 (0.36)	1.3 (0.04)	15.2 (0.40)
Winter	3.4 (0.09)	1.7 (0.04)	5.1 (0.13)

Percentages of the total habitat in each category are given in parentheses.
