# Supplementary material for: Historical range, extirpation and prospects for reintroduction of saigas in China
Source: Sci Rep. 2017 Mar 9;7:44200. doi: 10.1038/srep44200 (PMC5343587; doi:10.1038/srep44200)
Supplement: Supplementary Information [file srep44200-s1.pdf]

Supplementary information for:

Historical range, extirpation and prospects for reintroduction of saigas in China

Shaopeng Cui[1, 2], E. J. Milner-Gulland[3], Navinder J. Singh[4], Hongjun Chu[5, 6], Chunwang Li[1], Jing Chen[1, 2], and Zhigang Jiang[1, 2]*

[1] Key Laboratory of Animal Ecology and Conservation Biology, Institute of Zoology, Chinese Academy of Sciences, Beijing, China

[2] University of Chinese Academy of Sciences, Beijing, China

[3] Department of Zoology, University of Oxford, South Parks Road, Oxford, United Kingdom

[4] Department of Wildlife, Fish and Environmental Studies, Swedish University of Agricultural Sciences, Umeå, Sweden

[5] College of Resources and Environment Sciences, Xinjiang University, Urumqi, Xinjiang, China

[6] Altay Management Station, Mt. Kalamaili Ungulate Nature Reserve, Altay, Xinjiang, China

***Corresponding author**. E-mail: jiangzg@ioz.ac.cn

Supplemental material list:
Supplemental Figure S1
Supplemental Table S1
Supplemental Table S2
Supplemental references

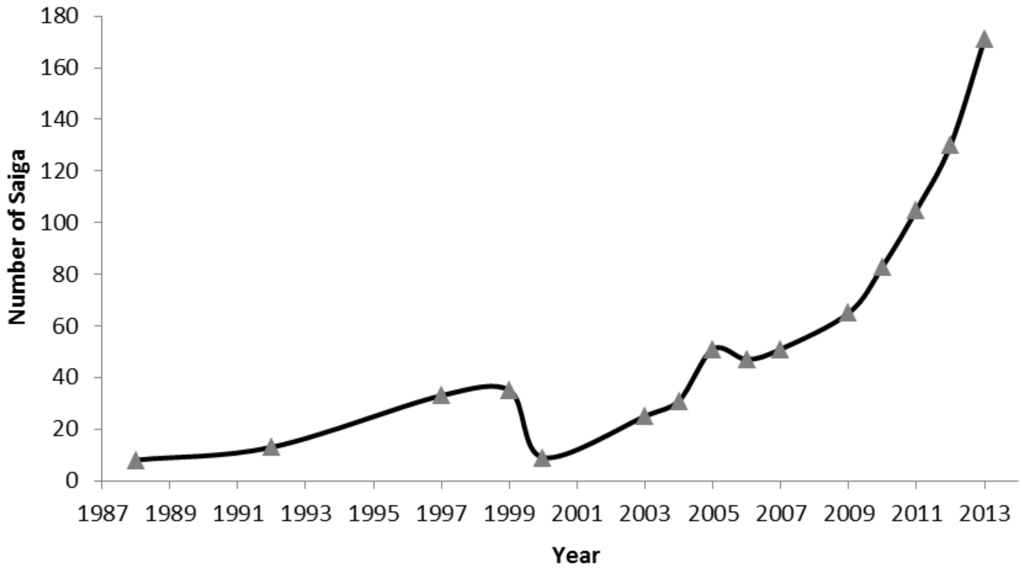

Supplementary Figure S1 Development of Saiga population size in Wuwei Endangered Wildlife Breeding Center.

Supplemental Table S1 Historical distribution records of saiga antelope (*Saiga tatarica*) in China.

Date	Location description	References
***c.* 1758-1846**	Around Hami City	Zhong, 1937[1] (p. 98)
***c.* 1908**	Shanshan County	He, 1993[2] (p. 268, the figure in p. 269; citing Chen, 1908[3])
***c.* 1918**	Wusu County	He, 1993[2] (p. 268, the figure in p. 269; citing Deng, 1918[4])
***c.* 1890-1922**	Manas County	He, 1993[2] (p. 268, the figure in p. 269; citing Wu, 1936[5])
***c.* 1890-1922**	Qitai County	He, 1993[2] (p. 268, the figure in p. 269; citing Wu, 1936[5])
***c.* 1890-1922**	Yining County	He, 1993[2] (p. 268, the figure in p. 269; citing Wu, 1936[5])
***c.* 1890-1922**	Buerjin County	He, 1993[2] (p. 268, the figure in p. 269; citing Wu, 1936[5])
***c.*1930**	Bole County	Zhang, 1997[6] (p. 130); Yang, 2006[7] (p. 436)
***c.* 1930**	Western Yumin county	Zhang, 1997[6] (p. 130); Yang, 2006[7] (p. 436)
***c.* 1900-1940**	Northern Beishan Mountains and western Inner Mongolia	Meng and Wang, 1999[8] (p. 2)
***c.* 1920-1940**	Western Hoboksar Mongol Autonomous County	Zhang, 1997[6] (p.131, Fig. 108; citing Bobrinskoy et al., 1944[9])
***c.* 1920-1940**	The bordering area between Western Fuhai County and Hoboksar Mongol Autonomous County	Zhang, 1997[6] (p.131, Fig. 108; citing Bobrinskoy et al., 1944[9])
***c.* 1920-1940**	Northern Jimunai County	Zhang, 1997[6] (p.131, Fig. 108; citing Bobrinskoy et al., 1944[9])
***c.* 1950-1960**	Around the Beitashan mountain	Wang *et al.* 1983[10] (p. 53, 55); Gao, 1991[11] (p. 123); Meng and Wang, 1999[8] (p. 2)
***c.* 1950-1960**	Alashankou	Liang, 1986[12] (p. 116); Gao, 1991[11] (p. 123); Wang *et al.*, 1997[13] (p. 309); Gao, 2005[14] (p. 208); Chen, 2006[15] (p. 77)
***c.* 1950-1960**	China-Kazakhstan Border areas in Jimunai County	Liang, 1986[12] (p. 116)
***c.* 1950-1960**	China–Mongolia border areas in	Liang, 1986[12] (p. 116)

	Mulei County	
***c.* 1950-1960**	Ili area	Sun, 1981[16] (p. 29); Xie *et al.*, 2004[17]; Yang, 2006[7] (p. 436)
***c.* 1950-1960**	China-Mongolia border areas in Yiwu County	Liu and Zhu, 1994[18] (p. 54)
***c.* 1950-1960**	China-Kazakhstan border areas in Yumin County and western Baerluke Mountain	Dang *et al.*, 1997[19] (p. 124); Xie *et al.*, 2004[17]; Abudoushalike, 2011[20] (p. 90);
***c.* 1950-1960**	China-Kazakhstan border areas in Tuoli County	Dang *et al.*, 1997[19] (p. 124)
***c.* 1950-1960**	Emin County	Li, 2000[21] (p. 94)
***c.* 1950-1960**	China-Mongolia border areas in Qitai County around Beitashan Mountain	Wang *et al.* 1983[10] (p. 52); Zhou and Li, 1994[22] (p. 72)
***c.* 1950-1960**	Around the Bortala River	Sun, 1981[16] (p. 29); Xie *et al.*, 2004[17]; Liu *et al.*, 1999[23] (p. 120)
***c.* 1950-1960**	China-Kazakhstan border areas in southern Habahe County	Wang *et al.*, 1983[10] (p. 55)
***c.* 1950-1960**	Southern Qinghe County	Wang *et al.*, 1983[10] (p. 55)
***c.* 1950-1960**	China-Mongolia border areas around northern Beishan Mountains	Zhang, 1986[24] (p. 30); Wang *et al.*, 1997[13] (p. 309); Meng and Wang, 1999[8] (p. 2)
***c.* 1950-1960**	Southern Fuyun County	Xie *et al.*, 2004[17]

Note: we removed the literature which described the distribution of Saiga in China very roughly (e.g. Przewalski[25] described that Saiga was distributed in the west of Junggar Basin). These references above included both primary and secondary sources.

Supplemental Table S2 Environmental variables used in species distribution models, with reasoning and source information.

Variable type	Code	Description	Why included	Source
Bioclimate	T_{mean}	Average monthly mean temperature	These factors are thought to be drivers of saiga migration and calving site selection[26-30]. In winter, levels of precipitation were chosen as a surrogate of snow depth which limits access to important winter resources[27,31].	WorldClim Version 1.4[32]
	T_{ran}	Mean monthly temperature range		
	$Prec_{mean}$	Average monthly mean precipitation		
Topography	Alt	Altitude	Saiga prefers open as well as flat habitat[27].	United States Geological Survey's Hydro1K dataset[33]
	CTI	Compound Topographic index		
Water availability	Dist2wat	Distance to the nearest water source	Water is an essential resource for saiga survival in the semi-arid rangelands[27]; distance from water is a key predictor of saiga distribution[28,30,34].	Global Lakes and Wetlands Database[35]
Human impact	HFI	Human Footprint Index	Saiga numbers have been greatly reduced due to considerable levels of poaching[27,36,37]. Saiga calving sites occur farther away from settlements than previously[28]. This variable was used as a proxy for anthropogenic disturbance.	Last of the Wild Data Version 2[38]

Supplemental References

1. Zhong, F. *Local Records of Hami in Xinjiang Province*. 97-98 (Chengwen Press, Taibei, 1937).
2. He, Y. *Historical Changes of Rare Mammals in China*. 267-269 (Hunan Science and Technology Press, Changsha, 1993).
3. Chen, G. *Local Records of Shanshan County*. (Sine nomine, 1908).
4. Deng, Z. *Local Records of Wusu County*. (Sine nomine, 1918).
5. Wu, T. *The Supplement to Xinjiang Chorography*. (Central Institute of Nationalities, Beijing, 1936).
6. Zhang, R. *Distribution of Mammalian Species in China*. 130-131 (China Forestry Publishing House, Beijing, 1997).
7. Yang, B. *Genetic Resources of Wild Artiodactyla and Perissodactyla in China*. 435-441 (Gansu Science and Technology Press, Lanzhou, 2006).
8. Meng, X. & Wang, W. Utilization and conservation of Saiga in China. *Chinese Wildlife* **5**, 2-5 (1999).
9. Bobrinskoy, N., Kuznetzov, B. & Kuzyakin, A. *Mammals of USSR*. (Sovetskaya Nauka, 1944).
10. Wang, T. *et al.* The animal resources of Altay and Beitashan Moutains in Xinjiang. *Chinese Wildlife* **3**, 52-55 (1983).
11. Gao, X. Animals for medicine in Xinjiang in *Studies on the Animals in Xinjiang* (ed Chinese Academy of Sciences Xinjiang Institute of Ecology and Geography) 122-134 (Science Press, Beijing 1991).
12. Liang, C. *Atlas of rare animals in Xinjiang*. 116-118 (China Forestry press, Beijing, 1986).
13. Wang, D., Luo, N., Gu, J. & Zhang, G. The introduction and domestication of Saiga (*Saiga tatarica*) in its original distribution area of China. *Chin. Biodivers.* **6**, 309-311 (1997).
14. Gao, X. *A Checklist on the Classification and Distribution of Vertebrate Species and Subspecies in Xinjiang*. 207-208 (Xinjiang Science and Technology Publishing House, Urumqi, 2005).
15. Chen, S. *Comprehensive Scientific Survey Report of Xia'erxili Nature Reserve in Xinjiang*. 77-94 (Xinjiang Science and Technology Publishing House, Urumqi, 2006).
16. Sun, X. Microscopic observation of antelope horns. *J. Chinese Med. Mater.* **4**, 29-31 (1981).
17. Xie, Y., Wang, S., He, F. & Zhao, E. *China Species Information System*. (2004) Available at: www.chinabiodiversity.com. (Accessed: 7th April 2012).
18. Liu, T. & Zhu, Z. *Local Records of Yiwu County*. 54-56 (Xinjiang University Press, Urumqi, 1994).
19. Dang, D., Chen, D. & Fan, S. *Local Chorographies of Tacheng Prefecture*. 124-126 (Xinjiang People's Publishing House, Urumqi, 1997).
20. Abudoushalike, N. *Comprehensive Scientific Survey Report of Baerluke Mountain Nature Reserve in Xinjiang*. 90-111 (Xinjiang University Press, Urumqi, 2011).
21. Li, R. *Local Records of Emin County*. 94-95 (Xinjiang People's Publishing House, Urumqi, 2000).
22. Zhou, H. & Li, Z. *Local Records of Qitai County*. 72-73 (Xinjiang University Press, Urumqi, 1994).
23. Liu, Z., Wang, W. & Li, S. *Local Chorographies of Bortala Mongol Autonomous Prefecture*. 120 (Xinjiang University Press, Urumqi, 1999).
24. Zhang, F. The animal resources in Gansu. *Chinese Wildlife* **4**, 29-31 (1986).
25. Przewalski, N. M. *Iz Zaisana cherez Khami v Tibet i na Verkhov'ia Zheltoi Reki [From Ziasan through Khami to Tibet and the Sources of the Yellow River]*. Vol. 1 37-41 (V.S. Balashev, 1883).
26. Milner-Gulland, E. J. A population model for the management of the saiga antelope. *J. Appl. Ecol.* **31**, 25-39, doi:10.2307/2404596 (1994).
27. Bekenov, A. B., Grachev, I. A. & Milner-Gulland, E. J. The ecology and management of the Saiga antelope in Kazakhstan. *Mamm. Rev.* **28**, 1-52, doi:10.1046/j.1365-2907.1998.281024.x (1998).
28. Singh, N. J., Grachev, I. A., Bekenov, A. B. & Milner-Gulland, E. J. Saiga antelope calving site

selection is increasingly driven by human disturbance. *Biol. Conserv.* **143**, 1770-1779, doi:10.1016/j.biocon.2010.04.026 (2010).

29. Singh, N. J., Grachev, I. A., Bekenov, A. B. & Milner-Gulland, E. J. Tracking greenery across a latitudinal gradient in central Asia – the migration of the saiga antelope. *Divers. Distrib.* **16**, 663-675, doi:10.1111/j.1472-4642.2010.00671.x (2010).
30. Singh, N. J. & Milner-Gulland, E. J. Conserving a moving target: planning protection for a migratory species as its distribution changes. *J. Appl. Ecol.* **48**, 35-46, doi:10.1111/j.1365-2664.2010.01905.x (2011).
31. Chilton, H. *The Where and Why of Saiga Antelope Distribution in West Kazakhstan*. (Imperial College London, London, 2011).
32. Hijmans, R. J., Cameron, S. E., Parra, J. L., Jones, P. G. & Jarvis, A. Very high resolution interpolated climate surfaces for global land areas. *Int. J. Climatol.* **25**, 1965-1978, doi:10.1002/joc.1276 (2005).
33. United States Geological Survey. *HYDRO1k, EROS (Earth Resources Observation System)*. (2002) Available at: http://edcdaac.usgs.gov/gtopo30/hydro/. (Accessed: 28th October 2013).
34. Milner-Gulland, E. J. A dynamic game model for the decision to join an aggregation. *Ecol. Model.* **145**, 85-99, doi:10.1016/s0304-3800(01)00381-7 (2001).
35. Lehner, B. & Döll, P. Development and validation of a global database of lakes, reservoirs and wetlands. *J. Hydrol.* **296**, 1-22, doi:10.1016/j.jhydrol.2004.03.028 (2004).
36. Milner-Gulland, E. J. *et al.* Dramatic declines in saiga antelope populations. *Oryx* **35**, 340-345 (2001).
37. Milner-Gulland, E. J., Bekenov, A. B. & Grachov, Y. A. The real threat to saiga antelopes. *Nature* **377**, 488-489, doi:10.1038/377488a0 (1995).
38. Sanderson, E. W. *et al.* The human footprint and the last of the wild. *Bioscience* **52**, 891-904 (2002).
